# First Results of Concurrent Chemoradiation with Two Courses of 5 × 25 mg/m^2^ Cisplatin for Locally Advanced Head and Neck Cancer

**DOI:** 10.3390/jpm13061006

**Published:** 2023-06-16

**Authors:** Inga Zwaan, Tamer Soror, Christian Idel, Ralph Pries, Karl L. Bruchhage, Samer G. Hakim, Nathan Y. Yu, Dirk Rades

**Affiliations:** 1 Department of Radiation Oncology, University of Lubeck, 23562 Lubeck, Germany; inga.zwaan@uksh.de (I.Z.); tamer.soror@uksh.de (T.S.); 2 Department of Oto-Rhino-Laryngology & Head and Neck Surgery, University of Lubeck, 23562 Lubeck, Germany; christian.idel@uksh.de (C.I.); ralph.pries@uksh.de (R.P.); karl-ludwig.bruchhage@uksh.de (K.L.B.); 3 Department of Oral and Maxillofacial Surgery, University of Lubeck, 23562 Lubeck, Germany; samer.hakim@uni-luebeck.de; 4 Department of Oral and Maxillofacial Surgery, MSH Medical School Hamburg, Schwerin Campus, 19055 Schwerin, Germany; 5 Department of Radiation Oncology, Mayo Clinic, Phoenix, AZ 85054, USA; yu.nathan@mayo.edu

**Keywords:** head and neck cancer, chemoradiation, cisplatin, cumulative dose, treatment outcomes, toxicities

## Abstract

Many patients with squamous cell carcinoma of the head and neck (SCCHN) receive cisplatin-based chemoradiation. Cisplatin 100 mg/m^2^ every three weeks is toxic and alternative cisplatin regimens are desired. Two courses of 20 mg/m^2^/day 1–5 (cumulative 200 mg/m^2^) were shown to be similarly effective and better tolerated than 100 mg/m^2^ every three weeks. Previous studies suggested that cumulative doses >200 mg/m^2^ may further improve outcomes. In this study, 10 patients (group A) receiving two courses of 25 mg/m^2^/day 1–5 (cumulative 250 mg/m^2^) in 2022 were retrospectively matched and compared to 98 patients (group B) receiving two courses of 20 mg/m^2^/day 1–5 or 25 mg/m^2^/day 1–4 (cumulative 200 mg/m^2^). Follow-up was limited to 12 months to avoid bias. Group A achieved non-significantly better 12-month loco-regional control (100% vs. 83%, *p* = 0.27) and metastases-free survival (100% vs. 88%, *p* = 0.38), and similar overall survival (89% vs. 88%, *p* = 0.90). No significant differences were found regarding toxicities, completion of chemotherapy, and interruption of radiotherapy. Given the limitations of this study, chemoradiation with two courses of 25 mg/m^2^/day 1–5 appears an option for carefully selected patients as a personalized treatment approach. Longer follow-up and a larger sample size are needed to properly define its role.

## 1. Introduction

Concurrent chemoradiation including cisplatin is a common treatment regimen for squamous cell carcinoma of the head and neck (SCCHN) [1]. Several cisplatin-based regimens are available, of which 100 mg/m^2^ of cisplatin alone given on radiotherapy days 1, 22, and 43 is most frequently used [1,2,3,4,5,6]. However, since this regimen is associated with considerable acute toxicities, many patients are unable to receive the third course and, thus, a cumulative dose of 300 mg/m^2^ [7]. Several studies have shown that a cumulative dose of at least 200 mg/m^2^ of cisplatin is desirable to achieve satisfying treatment outcomes, irrespective of the regimen [8,9,10,11]. Besides two courses of 100 mg/m^2^ of cisplatin, a cumulative dose of 200 mg/m^2^ can also be achieved with the administration of low-dose daily cisplatin, 30–40 mg/m^2^ weekly, or fractionated cisplatin with two courses of 5 × 20 mg/m^2^ or 4 × 25 mg/m^2^ [5,6,12,13]. In a previous study, 100 mg/m^2^ of cisplatin on radiotherapy days 1, 22, and 43 led to significantly better loco-regional control and overall survival than weekly administration of 30–40 mg/m^2^ but was associated with considerably greater toxicity [13]. These results were confirmed in a phase III non-inferiority trial published two years later, where 100 mg/m^2^ of cisplatin every three weeks resulted in better loco-regional control but was more toxic than 30 mg/m^2^ weekly [14]. In 2022, the results of an interim analysis of a randomized phase II/III non-inferiority trial comparing weekly cisplatin with 40 mg/m^2^ to 100 mg/m^2^ every three weeks were published [15]. Weekly cisplatin was not inferior with respect to overall survival and was associated with fewer adverse events. The consequences of increased chemotherapy-related toxicity may include interruption or even discontinuation of the radiotherapy course, which can lead to worse disease outcomes [6,7,16].

Further studies investigated alternative cisplatin regimens for concurrent chemoradiation of SCCHN to decrease toxicity [5,6,12,13,14]. In addition to weekly administration, two courses of 20 mg/m^2^/day 1–5 were tested. In a retrospective study of 230 patients with locally advanced SCCHN, three courses of 100 mg/m^2^ cisplatin were not superior regarding loco-regional control, metastases-free survival, and overall survival, but were associated with significantly more serious adverse events than two courses of fractionated cisplatin (20 mg/m^2^ on 5 days) [12]. Two courses of fractionated cisplatin may also be administered with 25 mg/m^2^ on four consecutive days, which was recently shown to be similarly effective [6].

Both cisplatin regimens, two courses of 20 mg/m^2^/day 1–5 and 25 mg/m^2^/day 1–4, result in a cumulative dose of 200 mg/m^2^. Although it is widely agreed that 200 mg/m^2^ is the minimum cumulative dose to be achieved, a few studies have found that doses beyond 200 mg/m^2^ may lead to even better outcomes [8,17,18]. One study found cumulative doses of 230–270 mg/m^2^ to be optimal [17]. These data led to the idea of increasing the cumulative dose of two courses of fractionated cisplatin from 200 mg/m^2^ to 250 mg/m^2^. This can be achieved with the administration of two courses of 25 mg/m^2^/day 1–5. We present the data of 10 patients treated with chemoradiation including cisplatin 25 mg/m^2^/day 1–5. They were matched to 98 patients receiving chemoradiation with 20 mg/m^2^/day 1–5 or 25 mg/m^2^/day 1–4 of cisplatin, considering twelve baseline characteristics. Both groups were compared for loco-regional control, metastases-free survival, overall survival, acute and late toxicities, completion of the planned chemotherapy, and interruption of the radiotherapy course.

## 2. Materials and Methods

The data of 108 patients who received cisplatin-based concurrent chemoradiation for SCCHN were retrospectively analyzed. The study was approved by the responsible local ethics committee (University of Lübeck, 21-034). Ten patients (group A) assigned to chemoradiation with two courses of 25 mg/m^2^/day 1–5 cisplatin every four weeks (cumulative dose 250 mg/m^2^) in 2022 were matched to 98 patients (group B) assigned to chemoradiation with two courses of cisplatin of 20 mg/m^2^/day 1–5 or 25 mg/m^2^/day 1–4 (cumulative dose 200 mg/m^2^) between 2012 and 2022 (97 patients until 2021). Planned total doses of external beam radiotherapy were either 70 Gy (2.0 Gy per fraction) for definitive treatment or 64-70 Gy (2.0 Gy per fraction) for adjuvant treatment and risk factors for local failure including incomplete resection, close margins, unclear extent of resection, and extracapsular spread of lymph node metastases. One patient in group A with initial pT1pN2b disease received external beam radiotherapy with 70 Gy following incomplete resection of a N2b recurrence. For the analyses, the patient was included in subgroups T1-2 and N2-3 (see baseline characteristics below). External beam radiotherapy was performed as volumetric modulated arc therapy (VMAT) with median 66 Gy. One patient in group A and six patients in group B received a brachytherapy boost of 3-5 fractions of 2.5–3.0 Gy (total 7.5–15.0 Gy) after 64–66 Gy of EBRT.

Groups A and B were compared for baseline characteristics, treatment outcomes (loco-regional control, metastases-free survival, overall survival), interruption of radiotherapy for more than one week, completion of chemotherapy, and toxicities. Baseline characteristics included age (≤63 years vs. ≥64 years, median age = 64 years), gender (female vs. male), Karnofsky performance score (KPS ≤ 80 vs. KPS 90–100), main tumor site (oropharynx/oral cavity vs. hypopharynx/larynx), primary tumor stage (T1−2 vs. T3−4), nodal stage (N0-1 vs. N2-3), histologic grade (G1-2 vs. G3), human papilloma virus (HPV) status (negative vs. positive), upfront surgery (no vs. yes), history of smoking prior to chemoradiation (no vs. yes), smoking during chemoradiation (no vs. yes), and pre-treatment hemoglobin level (<12 vs. ≥12 g/dL) (Table 1).

### Statistical Analyses

For the comparisons of group A and group B regarding the distributions of baseline characteristics, we used Fisher’s exact test. The same test was used for the comparisons regarding interruption of radiotherapy, completion of chemotherapy, and toxicities. Loco-regional control, metastases-free survival, and overall survival were calculated from the last day of radiotherapy. Since all patients in group A were treated in 2022 and 99% of the patients in group B were treated between 2012 and 2021, the maximum follow-up period of survivors was limited to 12 months to avoid a potential bias caused by the different lengths of follow-up. For univariable analyses of loco-regional control, metastases-free survival, and overall survival, we used the Kaplan–Meier method and the log-rank test. Characteristics significantly associated with outcomes (*p* < 0.05) were additionally included in a multivariable analysis, which was performed with a Cox proportional hazards model. These analyses were performed using the software BlueSky Statistics 10 GA (BlueSky Statistics LLC, Chicago, IL, USA).

## 3. Results

**Figure 1:**
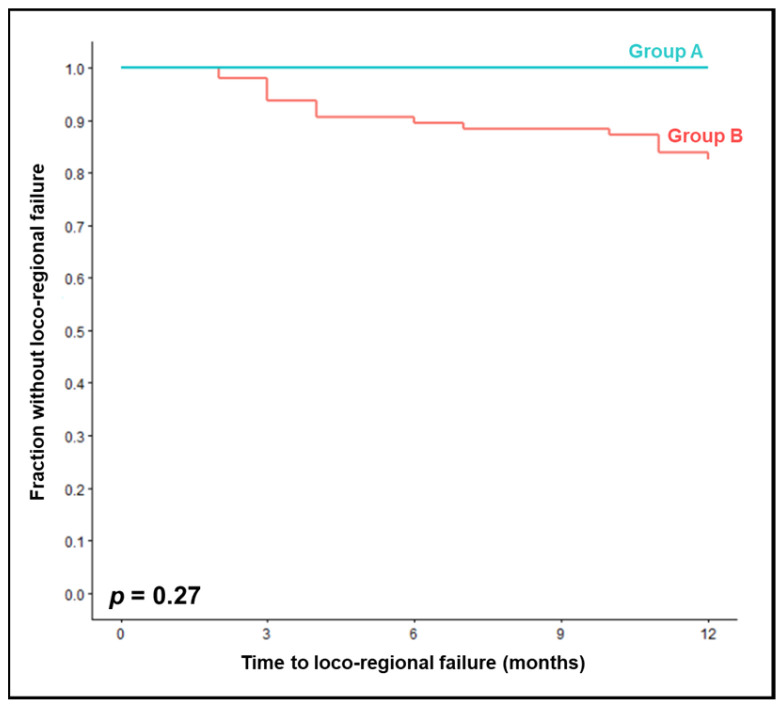
Comparison of chemoradiation with two courses of 25 mg/m^2^/day 1–5 cisplatin (group A) vs. two courses of 20 mg/m^2^/day 1–5 or 25 mg/m^2^/day 1–4 cisplatin (group B) for loco-regional control.

The cisplatin regimen used in group A, namely, two courses of 5 × 25 mg/m^2^, resulted in non-significantly better loco-regional control at 12 months than the regimens used in group B (100% vs. 83%, *p* = 0.27, Figure 1). On univariable analysis, improved loco-regional control was significantly associated with a KPS of 90–100 (*p* = 0.026), N-stage 0–1 (*p* = 0.013), and HPV-positivity (*p* = 0.009). Trends for better loco-regional control were found for T-stage 1–2 (*p* = 0.098), cancer of the oropharynx or oral cavity (*p* = 0.093), and pre-chemoradiation hemoglobin levels of ≥12 g/dL (*p* = 0.074). Relations between all the investigated baseline characteristics and loco-regional control are summarized in Table 2. In addition, completion of chemotherapy as planned showed a trend for better loco-regional control (*p* = 0.065). In the multivariable analysis considering the significant characteristics, HPV status showed a trend (hazard ratio [HR] 0.15, 95% confidence interval [CI] 0.02–1.25, *p* = 0.08). KPS (HR 0.42, 95% CI 0.08–2.08, *p* = 0.29) and nodal stage (HR 3.07, 95% CI 0.37–25.25, *p* = 0.30) were not significant in the multivariable analysis of loco-regional control.

**Figure 2:**
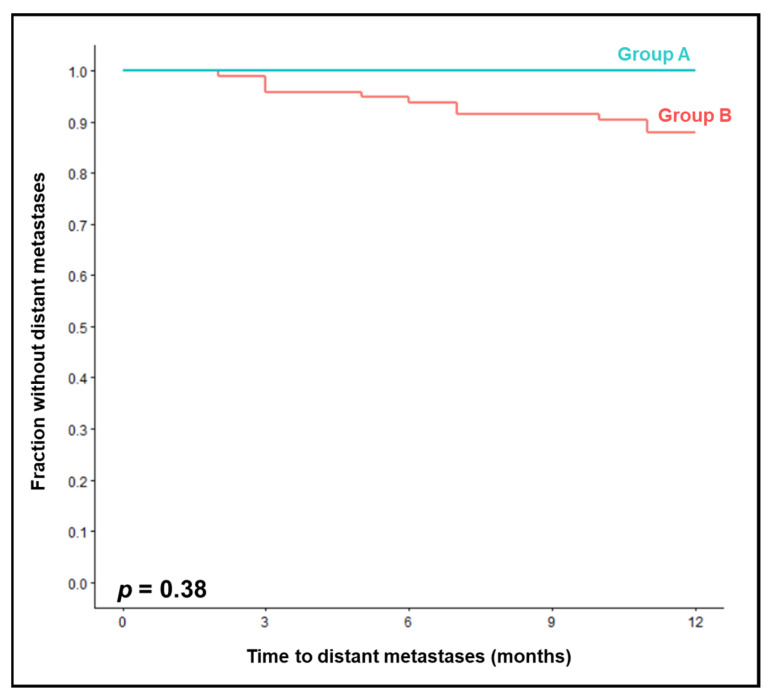
Comparison of chemoradiation with two courses of 25 mg/m^2^/day 1–5 cisplatin (group A) vs. two courses of 20 mg/m^2^/day 1–5 or 25 mg/m^2^/day 1-4 cisplatin (group B) for metastases-free survival.

In group A, metastases-free survival at 12 months was non-significantly better than in group B (100% vs. 88%, *p* = 0.38, Figure 2). On univariable analysis of metastases-free survival (Table 3), no characteristic was significantly associated with improved outcomes. Trends were found for a KPS of 90–100 (*p* = 0.094) and not smoking during chemoradiation (*p* = 0.073). Since no characteristic was significant on univariable analysis, a multivariable analysis was not performed. 

**Figure 3:**
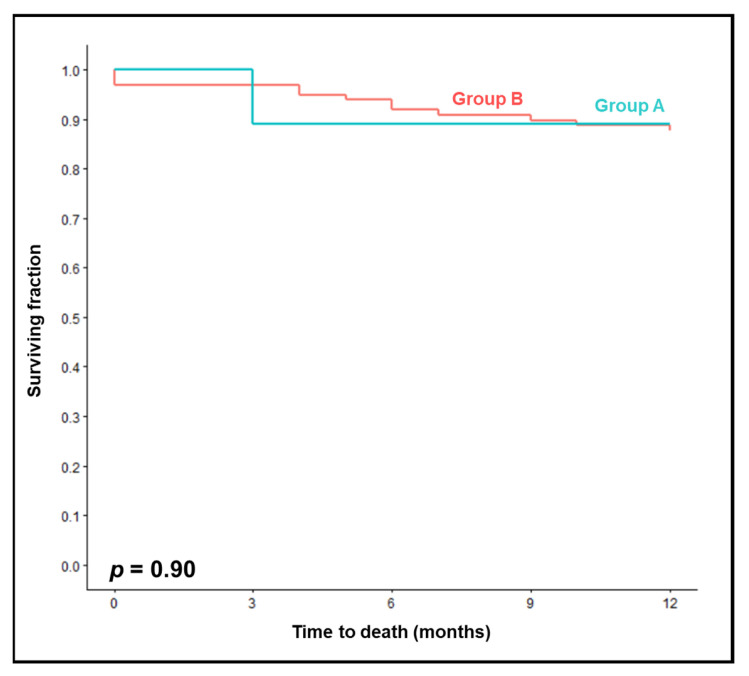
Comparison of chemoradiation with two courses of 25 mg/m^2^/day 1–5 cisplatin (group A) vs. two courses of 20 mg/m^2^/day 1–5 or 25 mg/m^2^/day 1-4 cisplatin (group B) for overall survival.

The 12-month overall survival rates were almost identical in group A and group B (89% vs. 88%, *p* = 0.90, Figure 3). On univariable analysis (Table 4), improved overall survival was significantly associated with pre-chemoradiation hemoglobin levels ≥ 12 g/dL (*p* = 0.014), and almost significantly with a KPS of 90–100 (*p* = 0.051). In the multivariable analysis of overall survival including only the hemoglobin level, this factor was significant (HR 0.26, 95% CI 0.08–0.83, *p* = 0.023). When including both characteristics, the hemoglobin level showed a trend (HR 0.33, 95% CI 0.10–1.11, *p* = 0.072), and KPS was not significant (HR 0.42, 95% CI 0.11–1.59, *p* = 0.20).

No significant differences were found between group A and group B with respect to acute and late toxicities in terms of grade ≥ 2 and grade ≥ 3 oral mucositis, grade ≥ 2 and grade ≥ 3 radiation dermatitis, grade ≥ 2 and grade ≥ 3 xerostomia, grade ≥ 2 and grade ≥ 3 cervical lymphedema, grade ≥ 2 and grade ≥ 3 nausea, grade ≥ 1 hearing problems, grade ≥ 1 and grade ≥ 2 decreased renal function, and grade ≥ 2, grade ≥ 3 and grade 4 hematotoxicity (Table 5). Chemotherapy was completed as planned by 50% of the patients in group A and 56% in group B (*p* = 0.75). At least 80% of the planned cisplatin dose was received by 80% and 70% of the patients, respectively (*p* = 0.72). In group A, 80% of the patients received a cumulative cisplatin dose of ≥225 mg/m^2^. The interruption of radiotherapy for more than one week occurred in 0% (group A) and 11% (group B) of the patients, respectively (*p* = 0.59).

## 4. Discussion

Standard chemoradiation for SCCHN including concurrent three-weekly cisplatin (100 mg/m^2^ on days 1, 22, and 43) is associated with significant toxicities [2,3,4,7]. Therefore, many patients are unable to complete the chemotherapy as planned. Two courses of 100 mg/m^2^ may be sufficient, since previous studies and a systematic review found that cumulative doses of 200 mg/m^2^ resulted in better outcomes than doses of less than 200 mg/m^2^ [8,9,10,11]. However, data suggest that cumulative doses greater than 200 mg/m^2^ may further improve the outcomes of chemoradiation for SCCHN, regardless of the cisplatin regimen applied [8,17,18].

In our study, we investigated the potential value of two courses of 5 × 25 mg/m^2^, resulting in a cumulative dose of 250 mg/m^2^. This regimen was chosen since it was close to the standard regimen used for the concurrent chemoradiation of SCCHN by several disciplines at our university hospital. This standard regimen includes a cumulative dose of 200 mg/m^2^, which is achieved by the administration of two courses of 5 × 20 mg/m^2^ or 4 × 25 mg/m^2^ [6]. This regimen became the standard after it was shown to be similarly effective but significantly less toxic compared to 100 mg/m^2^ of cisplatin given every three weeks in a previous study of 230 patients [12]. In that study, 5 × 20 mg/m^2^ resulted in similar 3-year loco-regional control (74% vs. 79%, *p* = 0.53) and metastases-free survival (79% vs. 74%, *p* = 0.67) and non-significantly better 3-year overall survival (80% vs. 68%, *p* = 0.14) [12]. Moreover, 5 × 20 mg/m^2^ was associated with significantly less pneumonia or sepsis (*p* = 0.003), grade ≥ 2 nausea (*p* < 0.001), grade ≥ 2 nephrotoxicity (*p* = 0.005), grade ≥ 2 xerostomia (*p* = 0.002), and grade ≥ 2 ototoxicity (*p* = 0.048).

We present the first results of ten patients who were assigned to concurrent chemoradiation including two courses of 5 × 25 mg/m^2^ cisplatin. To measure this regimen’s efficacy and toxicity, these ten patients were matched to a cohort of 98 patients who received chemoradiation with two courses of 5 × 20 mg/m^2^ or 4 × 25 mg/m^2^. Non-significantly more patients in group A smoked during chemoradiation (*p* = 0.31). Otherwise, both groups were well balanced with respect to another 11 baseline characteristics with *p*-values ranging between 0.74 and 1.00 (Table 1). Moreover, to reduce the risk of bias due to different follow-up times, we limited the follow-up to maximum 12 months in both groups. This was necessary because all patients in group A were treated in 2022 and all but one patient in group B were treated prior to 2022. At 12 months, chemoradiation with 5 × 25 mg/m^2^ of cisplatin resulted in loco-regional control rates and metastases-free survival rates of 100% each, which were both non-significantly better than in group B (83% and 88%, respectively). Remarkably, these advantages did not translate into improved 12-month overall survival. However, loco-regional control and metastases-free survival are important endpoints that both can significantly affect patients’ quality of life. The potential advantage of cumulative cisplatin doses greater than 200 mg/m^2^ was also found in previous studies [8,17,18]. In the retrospective study of Peng et al. that included a large cohort of patients with nasopharynx cancer, the risk of death did not significantly change until a cumulative cisplatin dose of 180 mg/m^2^, decreased considerably until a cumulative dose of 250 mg/m^2^, and increased again until 300 mg/m^2^ [17]. The authors considered cumulative doses of cisplatin between 230 and 270 mg/m^2^ optimal and recommended a cumulative dose of 240 mg/m^2^. Yang et al. compared radiotherapy alone to concurrent chemoradiation with cumulative cisplatin doses of ≤200 mg/m^2^ or >200 mg/m^2^ in patients with nasopharynx cancer [18]. Five-year failure-free survival rates were 70.4%, 74.4% and 82.6%, respectively (*p* < 0.03); and five-year overall survival rates were 79.5%. 83.8% and 90.8%, respectively (*p* < 0.01). Moreover, in the systematic review of Strojan et al., the cumulative cisplatin dose was positively associated with overall survival [8]. The authors stated that the recommended cumulative cisplatin dose during concurrent chemoradiation for SCCHN seemed to be at least 200 mg/m^2^. However, cumulative doses >200 mg/m^2^ (up to 270 mg/m^2^) appeared to lead to even better overall survival [8].

In addition to the favorable outcomes achieved with two courses of 5 × 25 mg/m^2^, this regimen was not associated with significantly increased toxicities compared to the regimes used in group B. The rates of Grade 3 non-hematological toxicities ranged between 0% and 20%, which are considerably lower rates when compared to standard chemoradiation of SCCHN with three-weekly 100 mg/m^2^ of cisplatin [2,3,4,12]. Moreover, 80% of the patients could receive cumulative doses of 225–250 mg/m^2^, and no patients required an interruption of the radiotherapy course for a week or longer. However, despite these promising data regarding the use of 5 × 25 mg/m^2^ of cisplatin, one should be aware of the limitations of the present study. These include the retrospective study design, the small number of patients included in group A, and the limited follow-up period of only 12 months. Long-term results in a larger cohort of patients are required to properly identify the value of this regimen. Depending on these results, prospective randomized trials may be designed that compare two courses of 5 × 25 mg/m^2^ of cisplatin to other cisplatin regimens, particularly to 100 mg/m^2^ of cisplatin given every three weeks. Despite its high toxicity profile, three-weekly cisplatin is the standard regimen for chemoradiation of SCCHN in many centers worldwide. Until then, two courses of 5 × 25 mg/m^2^ cisplatin may be considered for selected patients, preferably younger patients with a good performance status and a low comorbidity index.

## 5. Conclusions

Two courses of 25 mg/m^2^/day 1–5 cisplatin (cumulative 250 mg/m^2^) resulted in non-significantly better loco-regional control, non-significantly better metastases-free survival, and similar overall survival when compared to two courses of 20 mg/m^2^ on five days or 25 mg/m^2^ on four days (cumulative 200 mg/m^2^). Moreover, the higher-dose cisplatin regimen was not associated with significantly increased acute or late toxicities, a lower rate of completion of chemotherapy, or more interruptions of the radiotherapy course. Given the limitations of this study including the retrospective design, the small number of patients in the higher-dose group, and the short follow-up period, chemoradiation with two courses of 25 mg/m^2^/day 1–5 may be considered an alternative option for carefully selected patients with SCCHN requiring chemoradiation as a personalized treatment approach. However, a longer follow-up and a larger sample size are needed to properly define the role of this cisplatin regimen. Depending on the results of a more mature study, the regimen may be tested in a prospective randomized trial including larger groups of patients.

## Figures and Tables

**Table 1:** Distributions of baseline characteristics in patients scheduled for cumulative 250 mg/m^2^ of cisplatin (group A, *n* = 10) and patients scheduled for cumulative 200 mg/m^2^ of cisplatin (group B, *n* = 98).

Characteristic	Group A *n* Patients (%)	Group B *n* Patients (%)	*p*-Value
Age			0.74
≤63 years	4 (40)	49 (50)	
≥64 years	6 (60)	49 (50)	
Gender			1.00
Female	1 (10)	16 (16)	
Male	9 (90)	82 (84)	
Karnofsky performance status			0.75
≤80	6 (60)	52 (53)	
90–100	4 (40)	46 (47)	
Main tumor site			1.00
Oropharynx/oral cavity	7 (70)	72 (73)	
Hypopharynx/larynx	3 (30)	26 (27)	
Primary tumor stage			1.00
T1-2	4 (40)	36 (37)	
T3-4	6 (60)	62 (63)	
Nodal stage			1.00
N0-1	3 (30)	34 (35)	
N2-3	7 (70)	64 (65)	
Histologic grade ^a^			1.00
G1-2	5 (50)	46 (49)	
G3	5 (50)	47 (51)	
HPV status ^b^			1.00
Negative	4 (50)	36 (49)	
Positive	4 (50)	37 (51)	
Upfront surgery			1.00
No	4 (40)	44 (45)	
Yes	6 (60)	54 (55)	
Pre-treatment history of smoking ^c^			1.00
No	1 (10)	16 (18)	
Yes	9 (90)	73 (82)	
Smoking during chemoradiation ^d^			0.31
No	4 (40)	54 (60)	
Yes	6 (60)	36 (40)	
Pre-treatment hemoglobin level ^e^			0.74
<12 g/dL	3 (30)	39 (40)	
≥12 g/dL	7 (70)	58 (60)	

HPV: human papilloma virus. The *p*-values were calculated with Fisher’s exact test. ^a^ unknown in 5 patients; ^b^ unknown in 27 patients; ^c^ unknown in 9 patients; ^d^ unknown in 8 patients; ^e^ unknown in 1 patient.

**Table 2:** Loco-regional control related to type of chemotherapy and baseline characteristics (univariable analyses).

Characteristic	Loco-Regional Control (%)		*p*-Value
	At 6 Months	At 12 Months	
Planned cumulative cisplatin dose			0.27
250 mg/m^2^ (group A)	100	100	
200 mg/m^2^ (group B)	89	83	
Age			0.27
≤63 years	84	80	
≥64 years	96	87	
Gender			0.25
Female	94	94	
Male	90	82	
Karnofsky performance status			0.026
≤80	83	76	
90–100	98	92	
Main tumor site			0.093
Oropharynx/oral cavity	91	88	
Hypopharynx/larynx	89	72	
Primary tumor stage			0.098
T1-2	95	92	
T3-4	88	79	
Nodal stage			0.013
N0-1	97	97	
N2-3	87	77	
Histologic grade ^a^			0.60
G1-2	88	81	
G3	92	85	
HPV status ^b^			0.009
Negative	84	78	
Positive	100	97	
Upfront surgery			0.11
No	89	76	
Yes	91	89	
Pre-treatment history of smoking ^c^			0.73
No	93	86	
Yes	90	82	
Smoking during chemoradiation ^d^			0.27
No	93	87	
Yes	87	77	
Pre-treatment hemoglobin level ^e^			0.074
<12 g/dL	80	76	
≥12 g/dL	97	88	

HPV: human papilloma virus. The *p*-values were calculated with the log-rank test. ^a^ unknown in 5 patients; ^b^ unknown in 27 patients; ^c^ unknown in 9 patients; ^d^ unknown in 8 patients; ^e^ unknown in 1 patient.

**Table 3:** Metastases-free survival related to type of chemotherapy and baseline characteristics (univariable analyses).

Characteristic	Metastases-Free Survival (%)		*p*-Value
	At 6 Months	At 12 Months	
Planned cumulative cisplatin dose			0.38
250 mg/m^2^ (group A)	100	100	
200 mg/m^2^ (group B)	94	88	
Age			0.35
≤63 years	92	86	
≥64 years	96	92	
Gender			0.48
Female	94	94	
Male	94	87	
Karnofsky performance status			0.094
≤80	89	84	
90–100	100	94	
Main tumor site			0.14
Oropharynx/oral cavity	95	92	
Hypopharynx/larynx	93	80	
Primary tumor stage			0.16
T1-2	97	95	
T3-4	92	85	
Nodal stage			0.62
N0-1	100	90	
N2-3	91	88	
Histologic grade ^a^			0.77
G1-2	92	87	
G3	96	89	
HPV status ^b^			0.55
Negative	95	91	
Positive	100	94	
Upfront surgery			0.61
No	96	91	
Yes	93	87	
Pre-treatment history of smoking ^c^			0.66
No	100	85	
Yes	95	90	
Smoking during chemoradiation ^d^			0.073
No	100	94	
Yes	90	84	
Pre-treatment hemoglobin level ^e^			0.49
<12 g/dL	90	87	
≥12 g/dL	97	90	

HPV: human papilloma virus. The *p*-values were calculated with the log-rank test. ^a^ unknown in 5 patients; ^b^ unknown in 27 patients; ^c^ unknown in 9 patients; ^d^ unknown in 8 patients; ^e^ unknown in 1 patient.

**Table 4:** Overall survival related to type of chemotherapy and baseline characteristics (univariable analyses).

Characteristic	Overall Survival (%)		*p*-Value
	At 6 Months	At 12 Months	
Planned cumulative cisplatin dose			0.90
250 mg/m^2^ (group A)	89	89	
200 mg/m^2^ (group B)	92	88	
Age			0.17
≤63 years	92	92	
≥64 years	91	83	
Gender			0.11
Female	100	100	
Male	90	85	
Karnofsky performance status			0.051
≤80	86	82	
90–100	98	94	
Main tumor site			0.34
Oropharynx/oral cavity	90	86	
Hypopharynx/larynx	97	93	
Primary tumor stage			0.62
T1-2	95	89	
T3-4	90	86	
Nodal stage			0.71
N0-1	86	86	
N2-3	94	88	
Histologic grade ^a^			0.23
G1-2	94	92	
G3	90	84	
HPV status ^b^			0.11
Negative	85	80	
Positive	92	92	
Upfront surgery			0.89
No	90	87	
Yes	93	88	
Pre-treatment history of smoking ^c^			0.12
No	82	76	
Yes	93	90	
Smoking during chemoradiation ^d^			0.49
No	91	90	
Yes	90	84	
Pre-treatment hemoglobin level ^e^			0.014
<12 g/dL	83	78	
≥12 g/dL	97	94	

HPV: human papilloma virus. The *p*-values were calculated with the log-rank test. ^a^ unknown in 5 patients; ^b^ unknown in 27 patients; ^c^ unknown in 9 patients; ^d^ unknown in 8 patients; ^e^ unknown in 1 patient.

**Table 5:** Comparison of patients scheduled for cumulative 250 mg/m^2^ of cisplatin (group A) and patients scheduled for cumulative 200 mg/m^2^ of cisplatin (group B) with respect to acute and late toxicities.

Toxicity	Group A	Group B	*p*-Value
	*n* Patients (%)	*n* Patients (%)	
Oral mucositis ^a^			
Grade ≥ 2	5 (50)	70 (75)	0.13
Grade ≥ 3	2 (20)	24 (26)	1.00
Radiation dermatitis ^b^			
Grade ≥ 2	9 (90)	77 (84)	1.00
Grade ≥ 3	1 (10)	31 (34)	0.17
Xerostomia ^c^			
Grade ≥ 2	3 (30)	28 (29)	1.00
Grade ≥ 3	1 (10)	5 (5)	0.46
Cervical lymphedema ^d^			
Grade ≥ 2	1 (11)	10 (11)	1.00
Grade ≥ 3	0 (0)	0 (0)	1.00
Nausea ^e^			
Grade ≥ 2	0 (0)	13 (14)	0.60
Grade ≥ 3	0 (0)	2 (2)	1.00
Hearing problems ^f^			
Grade ≥ 1	2 (22)	21 (22)	1.00
Decreased renal function ^g^			
Grade ≥ 1	4 (40)	31 (32)	0.73
Grade ≥ 2	1 (10)	6 (6)	0.51
Hematotoxicity ^h^			
Grade ≥ 2	9 (90)	70 (73)	0.45
Grade ≥ 3	4 (40)	29 (30)	0.50
Grade 4	1 (10)	3 (3)	0.33

The *p*-values were calculated with Fisher’s exact test. ^a^ Unknown in 5 patients; ^b^ unknown in 6 patients; ^c^ unknown in 3 patients; ^d^ unknown in 5 patients; ^e^ unknown in 5 patients; ^f^ unknown in 2 patients; ^g^ unknown in 1 patient; ^h^ unknown in 2 patients.

## Data Availability

The data cannot be shared due to data protection regulations. Only evaluation of anonymized data is allowed according to the responsible ethics committee.
